# Association of the *PLCB1* gene with drug dependence

**DOI:** 10.1038/s41598-017-10207-2

**Published:** 2017-08-31

**Authors:** Judit Cabana-Domínguez, Carlos Roncero, Laura Pineda-Cirera, R. Felipe Palma-Álvarez, Elena Ros-Cucurull, Lara Grau-López, Abderaman Esojo, Miquel Casas, Concepció Arenas, Josep Antoni Ramos-Quiroga, Marta Ribasés, Noèlia Fernàndez-Castillo, Bru Cormand

**Affiliations:** 1 Departament de Genètica, Microbiologia i Estadística, Facultat de Biologia, Universitat de Barcelona, Barcelona, Catalonia Spain; 2 Centro de Investigación Biomédica en Red de Enfermedades Raras (CIBERER), Instituto de Salud Carlos III, Madrid, Spain; 3 Institut de Biomedicina de la Universitat de Barcelona (IBUB), Barcelona, Catalonia Spain; 4 Institut de Recerca Sant Joan de Déu (IR-SJD), Esplugues de Llobregat, Catalonia Spain; 5 Department of Psychiatry and Legal Medicine, Universitat Autònoma de Barcelona, Barcelona, Catalonia Spain; 6 Addiction and Dual Diagnosis Unit Vall Hebron, Psychiatric Services, Hospital Universitari Vall d’Hebron-ASPB, Barcelona, Catalonia Spain; 7 Biomedical Network Research Centre on Mental Health (CIBERSAM), Instituto de Salud Carlos III, Madrid, Spain; 8 Department of Psychiatry, Hospital Universitari Vall d’Hebron, Barcelona, Catalonia Spain; 9 Psychiatric Genetics Unit, Group of Psychiatry, Mental Health and Addiction, Vall d’Hebron Research Institute (VHIR), Universitat Autònoma de Barcelona, Barcelona, Catalonia Spain

## Abstract

Genetic factors involved in the susceptibility to drug addiction still remain largely unknown. MiRNAs seem to play key roles in the drug-induced plasticity of the brain that likely drives the emergence of addiction. In this work we explored the role of miRNAs in drug addiction. With this aim, we selected 62 SNPs located in the 3’UTR of target genes that are predicted to alter the binding of miRNA molecules and performed a case-control association study in a Spanish sample of 735 cases (mainly cocaine-dependent subjects with multiple drug dependencies) and 739 controls. We found an association between rs1047383 in the *PLCB1* gene and drug dependence that was replicated in an independent sample (663 cases and 667 controls). Then we selected 9 miRNAs predicted to bind the rs1047383 region, but none of them showed any effect on *PLCB1* expression. We also assessed two miRNAs binding a region that contains a SNP in linkage disequilibrium with rs1047383, but although one of them, hsa-miR-582, was found to downregulate *PLCB1*, no differences were observed between alleles. Finally, we explored the possibility that *PLCB1* expression is altered by cocaine and we observed a significant upregulation of the gene in the nucleus accumbens of cocaine abusers and in human dopaminergic-like neurons after cocaine treatment. Our results, together with previous studies, suggest that *PLCB1* participates in the susceptibility to drug dependence.

## Introduction

Drug dependence is one of the major health problems worldwide. In Europe, about 25% of adults are estimated to have tried illicit drugs at some point in their lives^1^. Usually drug consumers use more than one drug at the same time: for example, within the group of European individuals who consumed a psychoactive substance in the last 12 months, 33% had consumed two different substances and 10% had used three^2^. This high prevalence of polydrug abuse is due to common and drug-specific genetic and environmental factors^3–5^. It is well known that addictions are moderately to highly heritable (from 0.39 in the case of hallucinogens to 0.72 for cocaine), although the specific genetic risk factors involved in its predisposition remain largely unknown^6–9^. Transcriptomic studies in animal and cellular models, as well as human studies in postmortem brain samples from addicted individuals, have revealed that both acute and chronic drug exposure produce epigenetic adaptations and changes in gene expression^10^. Furthermore, recent studies have shown that some genes whose expression is altered by cocaine also contribute to cocaine dependence susceptibility^11, 12^.

MicroRNAs (miRNAs) are small regulatory noncoding RNA molecules (about 18–25 nucleotides in length) that control gene expression through direct binding to 3′untranslated regions (3′UTRs) of target mRNAs causing translational repression or mRNA degradation. One single miRNA can target and regulate hundreds of mRNAs and, conversely, one mRNA can be regulated by several miRNAs. This is a complex and dynamic system that allows the cells to fine-tune gene expression^13–15^.

MiRNAs are very abundant in the central nervous system and play important roles in neuronal development, differentiation and survival^16, 17^. Many studies have shown their contribution to several psychiatric disorders such as schizophrenia, bipolar disorder, autism or drug dependence^18–21^. In human prefrontal cortex of alcoholic patients 35 miRNA were found up-regulated as compared to controls^22^. Animal model studies have demonstrated that drugs of abuse induce robust alterations in the expression of a wide range of miRNAs. Cocaine administration in rats alters miR-124, miR-181 and let-7 in mesolimbic dopaminergic system^23, 24^ and miR-212 in dorsal striatum^25, 26^. On the other hand, alcohol regulates miR-9 increasing alcohol tolerance^27^. Also, miRNAs have been shown to play an important role in different processes related to addiction such as reward, synaptic plasticity, learning, memory, withdrawal and relapse^28^.

Some studies suggest that single nucleotide polymorphisms (SNPs) located in miRNAs or in their target sites can alter the miRNA-mediated regulation of gene expression that underlies disease and non-pathological phenotypes^29–31^. A recent study generated a transcriptome-wide map of the miRNA binding sites in human brain. Based on the interaction between argonaute 2 protein (AGO2) and miRNAs, they identify target regions in mRNAs. These regions contain 916 common SNPs that could potentially alter miRNA:mRNA binding^32^.

In this study, we aimed at examining the contribution to drug dependence susceptibility of SNPs that alter the binding of miRNAs to their target mRNAs. For that purpose we selected SNPs located in the 3’UTR identified in the study mentioned above and performed a case-control association study in drug addiction in a discovery and a replication samples from Spain. The identified variants were subjected to functional testing. Finally, we assessed the impact of cocaine on the expression of those genes where the associated SNPs are located.

## Material and Methods

### Association study

#### Subjects

**Figure 1:**
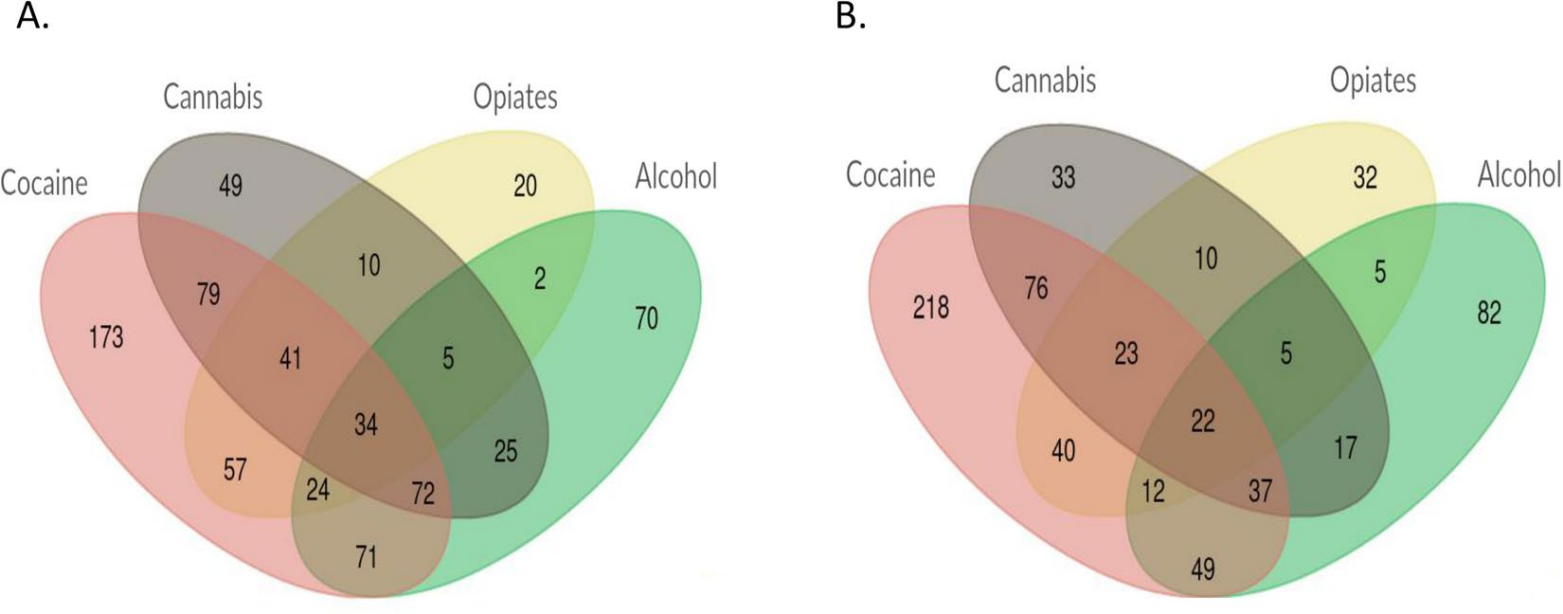
Distribution of the four main dependencies (cocaine, cannabis, alcohol and opiates) in the sample of patients included in the case-control association study, depicted in Venn diagrams. Other dependencies with a frequency lower than 10% are not displayed. (**A**) Discovery sample. (**B**) Replication sample.

Patients were recruited and evaluated at the Addiction and Dual Diagnosis Unit of the Psychiatry Department of the Hospital Universitari Vall d’Hebron (Barcelona, Spain) according to DSM-IV-TR criteria (Diagnostic and Statistical Manual of Mental Disorders, 4th ed. Text revision). The Structured Clinical Interview (SCID)^33^ was administered and volunteers with DSM-IV lifetime diagnosis for substance dependence were included in the study. About 73.5% of our patient sample consists of cocaine-dependent patients, most of which are dependent to other drugs of abuse. Controls were recruited at the Blood and Tissues Bank of Barcelona, and both patients and controls were Spanish and Caucasian, with the two last names (one from each parent) of Spanish origin. Other ethnicities such as Moroccan, Gypsies or South American individuals, among others, were discarded. Patients and controls were divided randomly into a discovery sample, which consisted of 735 patients and 739 controls, and a replication sample of 663 patients and 667 controls (Table 1 and Fig. 1). Population stratification was previously discarded in our sample^34^. The study was approved by the ethics committee of our institution, the Institutional Review Board of the University of Barcelona (IRB00003099), and informed consent was obtained from all participants, in accordance with the Helsinki Declaration. All experiments were performed in accordance with relevant guidelines and regulations.

**Table 1:** Descriptive characteristics of the Spanish Caucasian individuals with drug dependence and controls used in the case-control association study.

Gender N (%)	Discovery sample		Replication sample	
	Drug dependence N = 735	Control N = 739	Drug dependence N = 663	Control N = 667
Male	574 (78.1)	577 (78.1)	507 (76.5)	510 (76.5)
Female	161 (21.9)	162 (21.9)	156 (23.5)	157 (23.5)
**Age (mean and SD)**				
	37.3 ± 9.6	55.4 ± 15.9	37.3 ± 9.9	55.8 ± 16.3

SD: Standard deviation.

#### DNA isolation and quantification

Genomic DNA samples were obtained from peripheral blood lymphocytes using the salting-out method^35^ and were quantified using Nanodrop ND-1000 Spectrophotometer (Nanodrop Technologies, Thermo Fisher Scientific Inc., Wilmington, DE, USA).

#### SNP selection and genotyping

We selected SNPs in target genes that might alter the binding of regulator miRNAs using a previously described list of 916 SNPs located in AGO2 binding sites^32^. From this list we selected SNPs within the 3′UTR of genes and with a minimum allele frequency (MAF) of 0.15 in CEU individuals from HapMap project (www.hapmap.org; release 23). A total of 62 SNPs were selected under these criteria and genotyped in the discovery sample. We considered a maximum percentage of 5% of missing genotypes, and the SNPs finally evaluated had an average call rate of 99.6%. Nominally significant associated SNPs were subsequently assessed in a replication sample. Genotyping of both discovery and replication samples was performed by KASP technology (LGC, Teddington, Middlesex, UK).

#### Statistical analyses

The minimal statistical power for discovery (23–93%) and replication samples (18–81%) were calculated *post hoc* using the software Power Calculator for Genetic Studies (http://sph.umich.edu/csg/abecasis/CaTS/), under the multiplicative model (equivalent to log-additive) and assuming an odds ratio (OR) between 1.1 and 1.3, a significance threshold of 0.05, the lowest MAF value in our study (0.119), and a prevalence for substance dependence of 0.026^36^. The R library *SNPassoc* ^37^ was used to assess Hardy-Weinberg equilibrium (HWE, threshold set at *P* < 0.01) and to compare genotypic frequencies between cases and controls for each marker considering the log-additive model and a significance threshold of 0.05. As age differed significantly between cases and controls, we considered it as a covariate in all tests. All the p-values shown in the different association analyses are the ones adjusted for age, except in Table 2 and Supplementary Tables where both p-values are shown. The Bonferroni correction threshold for multiple testing was set at *P* < 8.5e-04 (0.05/59 SNPs) in the discovery sample, and at *P* < 7.2e-03 (0.05/7 SNPs) in the replication sample and in the pooled analysis.

**Table 2:** SNPs located in miRNA binding sites associated with drug dependence.

Marker	Locus	Discovery (735 cases–739 controls)				Replication (663 cases–667 controls)				Pooled analysis (1393 cases–1406 controls)		
		Alleles	p-value^1^	Adj. p-value^2^	OR [95% CI]^2^	Alleles	p-value^1^	Adj. p-value^2^	OR [95% CI]^2^	p-value^1^	Adj. p-value^2^	OR [95% CI]^2^
rs6840	*SCD5*	C/T	0.011	0.025	1.24 [1.03–1.50]	—	—	—		—	—	—
rs1285	*IDI1*	C/T	0.029	—		C/T	0.010	0.034	1.32 [1.02–1.71]	—	—	—
rs1872353	*FBXO45*	C/T	0.044	—		—	—	—		—	—	—
rs6855973	*GRIA2*	A/T	0.014	—		—	—	—		—	—	—
rs1047383	*PLCB1*	C/T	0.039	9.6e-03	1.27 [1.06–1.53]	C/T	3.8e-03	1.6e-03	1.37 [1.13–1.67]	4.8e-04	3.7e-04	1.29 [1.12–1.49]
rs1057377	*SPOCK3*	A/G	0.026	0.033	1.23 [1.02–1.49]*	—	—	—		—	—	—
rs2597775	*QDPR*	C/T	0.043	—		—	—	—		—	—	—

^1^Log-additive model;^2^Ajusted by age; Risk allele underlined.*When OR <1 the inverted score is shown; SNP, Single Nucleotide Polymorphism.

### Evaluation of functional effect of associated variants

#### Linkage disequilibrium analysis

Genotye data for the *PLCB1* gene plus 10 kb flanking sequences upstream and downstream were available for 554 individuals from our control sample^38^. The analysis of linkage disequilibrium (LD) was performed using Haploview software^39^ setting a maximum r^2^ threshold at 0.85.

#### Functional evaluation of SNPs effect on microRNA regulation

We assessed the possible functional effect of rs1047383 and two other variants found in LD with it (rs708910 and rs1047381), all located in the 3’UTR of the *PLCB1* gene. To do that, we used a luciferase reporter system to test the possible impact of these SNPs on the regulation of gene expression mediated by miRNAs, as previously described^12^. The prediction tools FuncPred, mirWalk, mirSNP, mrSNP, mirdSNP, miRNASNP and RNAhybrid were used to select miRNAs which binding sites in *PLCB1* is potentially affected by these SNPs. We chose the best predictions for each SNP: for rs1047383, hsa-miR-124-1, hsa-miR-139, hsa-miR-140, hsa-miR-144, hsa-miR-377, hsa-miR-506, has-miR-548h, hsa-miR-1324 and hsa-mir-3148; and for rs708910, hsa-miR-582 and hsa-miR-140. All miRNAs were cloned into a pCMV-MIR vector (OriGene, Rockville, MD, USA) and expression was confirmed after transfection into HeLa or HEK293 cells by qRT-PCR using the miScript PCR System (Qiagen, Hilden, Germany). Two regions from the 3’UTR of the *PLCB1* gene were cloned in the pmirGLO Dual-Luciferase miRNA Target Expression Vector (Promega, Madison, WI, USA): one fragment of the 3’UTR of the *PLCB1* gene containing SNP rs1047383 (hg19/chr20:8,864,935–8,865,116; 182 bp) and another one containing SNPs rs708910 and rs1047381 (hg19/chr20:8,864,108–8,864,373; 267 bp). All the constructs were used to test the effect of the selected miRNAs in HeLa or in HEK293 cells. Luciferase expression was assessed using the Dual-luciferase Reporter Assay System (Promega). As our data did not follow a normal distribution (tested using Shapiro-Wilk test), differences between the two conditions were evaluated with the nonparametric Mann-Whitney U-test using the SPSS statistics software version 22.0 (IBM, Armonk, NY, USA), and *P* < 0.05 was considered significant.

### Effect of cocaine on *PLCB1* expression

To assess the possible impact of cocaine on *PLCB1* expression we used data available from previous studies. Data from nucleus accumbens samples of human cocaine abusers (10 cases and 10 controls matched by age, race, sex and brain pH) were kindly provided by the authors^40^. When comparing differences between the expression levels of *PLCB1* between cases and controls, we considered the ratio between each case with its matched control. Normality of ratio was confirmed by using Shapiro-Wilk test (*P* = 0.91). Thus, the null hypothesis that the ratio is one, was tested with the parametric Student’s t test, using the SPSS statistics software version 22.0 and considering *P* < 0.05 as significant. Furthermore, we also used RNA samples obtained in a previous study from our group on the effect of cocaine on gene expression^12^. In this study we generated a human dopaminergic neuron-like model (differentiated SH-SY5Y cells) and RNA samples were obtained at different time points after an acute cocaine exposure (30 min). Samples were retrotranscribed using High-Capacity cDNA Reverse Transcription kit (Thermo Fisher Scientific). For the present study, we assessed *PLCB1* expression by quantitative Real-Time PCR (qRT-PCR) using LightCycler® 480 SYBR Green I Master (Roche Life Sciences, Branford, CT, USA), and relative quantification was performed as previously described, using *GAPDH* and *ACTB* as reference genes^12^. As we only have three replicates for each condition, differences between conditions were evaluated with a t-student test^41^ using SPSS statistics software version 22.0.

### Ethics statement

This study was approved by the local Ethics Committee and informed consent was obtained from all adult subjects, children and their parents according to the Helsinki declaration.

## Results

In this study we evaluated the contribution to drug dependence predisposition of SNPs located in the 3’UTR of genes expressed in the brain that are predicted to alter the binding of miRNA molecules.

We performed a case-control association study in a sample of 735 drug-dependent patients and 739 sex-matched controls from Spain. A total of 59 SNPs in 56 genes were finally evaluated (from the 62 SNPs that were genotyped, one showed poor genotyping and two were not in HWE). The comparison of genotype frequencies between cases and controls under the log-additive model showed nominally significant differences before adjusting by age for seven SNPs located in the genes *SCD5*, *IDI1*, *FBXO45*, *GRIA2*, *PLCB1*, *SPOCK3* and *QDPR* (Table 2 and Supplementary Table S1). These associated SNPs were subsequently evaluated in an independent Spanish sample of 663 drug-dependent patients and 667 sex-matched controls and the association remained significant for rs1285 in the *IDI1* gene (*P* = 0.034; OR = 1.32, CI = [1.02–1.71]) and rs1047383 in the *PLCB1* gene (*P* = 1.6e-03; OR = 1.37, CI = [1.13–1.67]). In the first one the direction of the effect was not the same in the discovery and replication samples, but it was so for the rs1047383 association, which also survived the Bonferroni correction (Table 2 and Supplementary Table S2). In the pooled analysis of both the discovery and replication samples, only rs1047383 in the *PLCB1* gene remained associated with the disorder (*P* = 3.7e-04; OR = 1.29, CI = [1.12–1.49], Table 2 and Supplementary Table S3), with a higher frequency of subjects carrying the C allele in the group of drug-dependent subjects (39%) as compared to controls (35%). This association withstood the Bonferroni correction for multiple testing. Finally, we also explored these results in the subgroup of cocaine-dependent patients, including patients where cocaine is one of multiple drugs of abuse or the only one (about 75% of our patients’ sample), and the association remained significant both in the discovery sample (551 cases; *P* = 0.03; OR = 1.25, CI = [1.02–1.53]) and in the replication sample (478 cases; *P* = 0.01; OR = 1.35, CI = [1.05–1.73]), and also in the pooled analysis (1,029 cases; *P* = 5.7e-03; OR = 1.26, CI = [1.07–1.48]).

We selected nine different miRNAs that potentially target the 3’UTR of *PLCB1*, with their binding being predicted to be affected by variation in rs1047383. No regulatory effect on *PLCB1* expression was observed for any of the assayed miRNAs using a gene reporter system in HeLa cells. We then investigated the existence of SNPs in LD with rs1047383 and found two polymorphisms, rs6056229 and rs708910 (r^2^ = 1 and 0.88, respectively). The first one is located outside the *PLCB1* gene and we could not find any functional prediction for it. The latter is located within the 3’UTR of *PLCB1* and is predicted to alter a binding site for several miRNAs by the FuncPred software. This SNP is only 25 bp distant from another SNP, rs1047381, which is in moderate LD with the associated variant in our Spanish sample (r^2^ = 0.61). To test this, we used a construct that includes both SNPs and assessed the possible effect of the two most frequent haplotypes (rs1047381C-rs708910G and rs1047381T-rs708910A) on the regulation mediated by hsa-miR-140 and hsa-miR-582. No differences were observed in HeLa cells, but in this cell line hsa-miR-582 could not be successfully overexpressed upon transfection. For this reason we repeated the experiment in HEK293 and observed that hsa-miR-582 decreases *PLCB1*expression (25%, *P* = 3.96e-03, Supplementary Figure 2), but without differences between the two rs1047381-rs708910 haplotypes.

**Figure 2:**
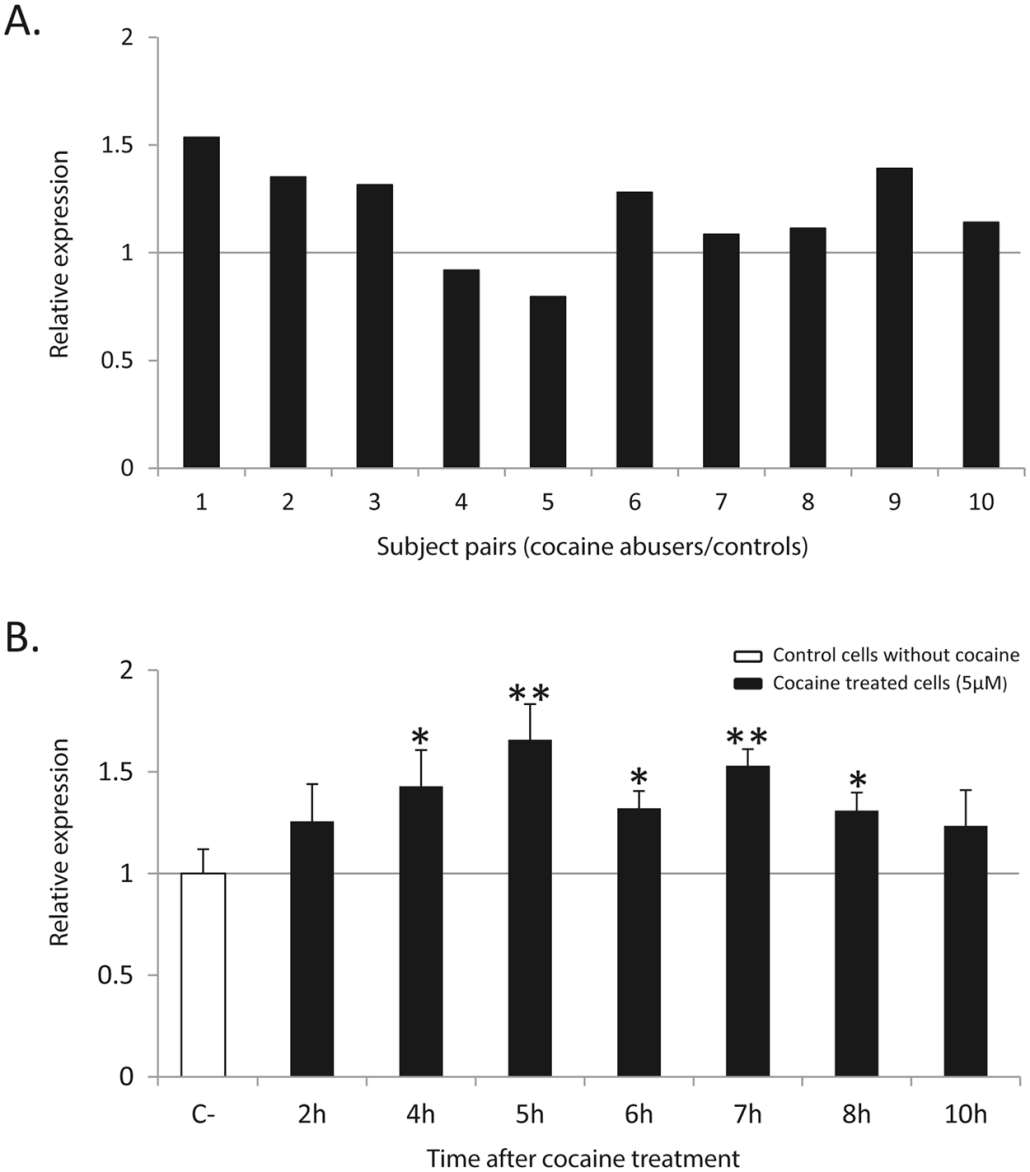
Expression of the *PLCB1* gene after treatment with cocaine. (**A**) Expression levels in the nucleus accumbens of cocaine abusers compared with their matched controls. (**B**) Transcription levels in human dopaminergic neuron-like cells (differentiated SH-SY5Y) at different time points after a 30-min exposure to 5 μM cocaine. Significant differences compared to control cells (not exposed to cocaine) normalized to *GAPDH* are indicated. Error bars indicate s.d. *p-value < 0.05, **p-value < 0.01.

Previous studies had reported that two genes (*NFAT5 *and *NTNG1*) with altered expression under cocaine also bear variants that confer susceptibility to cocaine dependence as shown by association studies^11, 12^. Since we have shown that *PLCB1* is involved in the vulnerability to cocaine dependence, we explored the possibility that its expression is altered by the action of cocaine. For that purpose we used data from a previous study using samples from nucleus accumbens of human cocaine abusers^40^. The sample’s mean expression levels of *PLCB1* gene were increased by 1.19-fold (*P* = 0.012) when we compared each case with its matched control. Furthermore, we investigated the effect of cocaine on the regulation of *PLCB1* in a human neuron-like dopaminergic model (differentiated SH-SY5Y cells) using samples previously produced in our lab^12^. We have now performed qRT-PCR experiments in this model and, interestingly, a significant upregulation of *PLCB1* after cocaine treatment was observed, showing a maximum of 1.65-fold increase (*P* = 5.8e-03) as compared to untreated cells (Fig. 2).

## Discussion

In this study we explored, for the first time, the possible role in drug dependence of SNPs located in the 3’UTR of genes potentially altering the binding of the corresponding mRNA to miRNA molecules. These SNPs were selected on the basis of a previous study^32^ which listed variants located in binding sites for the AGO2 protein, one of the molecules of the RNA-induced silencing complex (RISC) that interacts with both the miRNA and the mRNA. We found that the rs1047383 variant in the *PLCB1* gene is associated with drug dependence in two independent samples. Then we investigated a possible effect of this SNP and rs1047381-rs708910 (in LD with rs1047383) on miRNA binding, with negative results for all the tested miRNAs (a total of 10 out of 35 predictions identified in the databases we used). The only exception was hsa-miR-582, which reduced gene expression but with no differences between the two rs1047381-rs708910 haploalleles. It is important to note that, although many prediction tools are available, the degree of overlap of the different outputs is often limited^14^, so ranking the predictions is not straightforward.

The *PLCB1* gene encodes the Phospholipase C beta 1 protein expressed in the brain, mainly in cortex, hippocampus and amygdala. It is considered a molecular mediator of synaptic plasticity and it plays an important role in modulating cognitive behavior and emotions^42, 43^. Many neurotransmitters such as dopamine, serotonin and glutamate activate PLCβ1 by a G-protein-coupled receptor that signals through G_q/11_^44–47^. Furthermore, *PLCB1* has previously been related to other psychiatric and neurological disorders such as schizophrenia, autism and epilepsy^48–52^.

Several lines of evidence support a role for *PLCB1* in drug dependence. A region of overlapping clusters of SNPs in the *PLCB1* gene were identified in a previous study that assessed common genomic regions in two GWAS of illegal substance dependence and cocaine dependence^53^. However, in another GWAS reported by Gelernter *et al*. in 2014^54^ none of the *PLCB1* SNPs showed a suggestive association (*P* < 1e-05) with cocaine dependence. These discordant results might be explained by differences in the case-control designs, as the controls used in their study^54^ were individuals not dependent to cocaine who had taken this drug at least once in their lives and, in our study, the controls were individuals from the general population. Also, increased *Plcb1* expression was previously described in the nucleus accumbens of mice after administration of cocaine during 7 days and also during withdrawal^55^. This is consistent with our results, in which we identified increased expression in both the nucleus accumbens of human cocaine abusers and in cultured dopaminergic-like human neurons treated with cocaine. Furthermore, a previous study of our group identified changes in the expression of *PLCB1* in a mouse model of frustrated expected reward^56^.

In conclusion, although we could not prove that the SNP found associated with the phenotype alters miRNA-mediated regulation of gene expression, our data provide evidence for the contribution of the *PLCB1* gene to cocaine dependence, identifying an associated variant that was replicated in a second sample, as well as alterations in the expression of *PLCB1* induced by cocaine.

Several strengths and limitations of the present study should be discussed. Strengths: i) To minimize sample heterogeneity, both patients and controls in the discovery and replication samples were recruited in the same small geographical area around Barcelona (Spain); cases and controls were Spanish, Caucasian and sex-matched; ii) cases were evaluated by following a unique clinical assessment; iii) the associated variant was replicated in a second independent sample; iv) altered expression of *PLCB1* induced by cocaine was seen in human brain post-mortem samples and also in a human neuron-like model, with the same direction; v) previous studies are in agreement with our findings at *PLCB1* both in the association and expression studies. Limitations: i) The disease phenotype was not excluded in the control samples, which may have potentially diluted positive findings in the association study; ii) the sample size, 1398 cases and 1406 controls, is relatively small and may have led to false negative results; iii) the associated variant could not be proven to have a functional effect on the binding of any of the tested miRNAs, although it could have an effect on other non-investigated miRNAs; iv) changes in *PLCB1* expression levels could be proven at mRNA level but we could not test the protein as samples were not available for this type of analysis.

In conclusion, a variant in the *PLCB1* gene was found associated with drug dependence in two independent samples. Although the sample size is altogether relatively small, the results obtained in the discovery sample have been replicated. Also, the expression of *PLCB1* was found to be altered by cocaine. Together with previous results, this study highlights *PLCB1* as a gene that may contribute to drug dependence. Finally, these findings and previous results from our and other groups on *NFAT5* and *NTNG1* ^11, 12^ suggest that genes which expression is altered by the effect of drugs of abuse may play an important role in the susceptibility to drug dependence.

## Electronic supplementary material

Supplementary Information
