# Supplementary material for: Association of the PLCB1 gene with drug dependence
Source: Sci Rep. 2017 Aug 31;7:10110. doi: 10.1038/s41598-017-10207-2 (PMC5579249; doi:10.1038/s41598-017-10207-2)
Supplement: Supplementary file 1 — Supplementary Information [file 41598_2017_10207_MOESM1_ESM.pdf]

Association of the *PLCB1* gene with drug dependence

Judit Cabana-Domínguez, Carlos Roncero, Laura Pineda-Cirera, R. Felipe Palma-Álvarez, Elena Ros-Cucurull, Lara Grau-López, Abderaman Esojo, Miquel Casas, Concepció Arenas, Josep Antoni Ramos-Quiroga, Marta Ribasés, Noèlia Fernàndez-Castillo, Bru Cormand

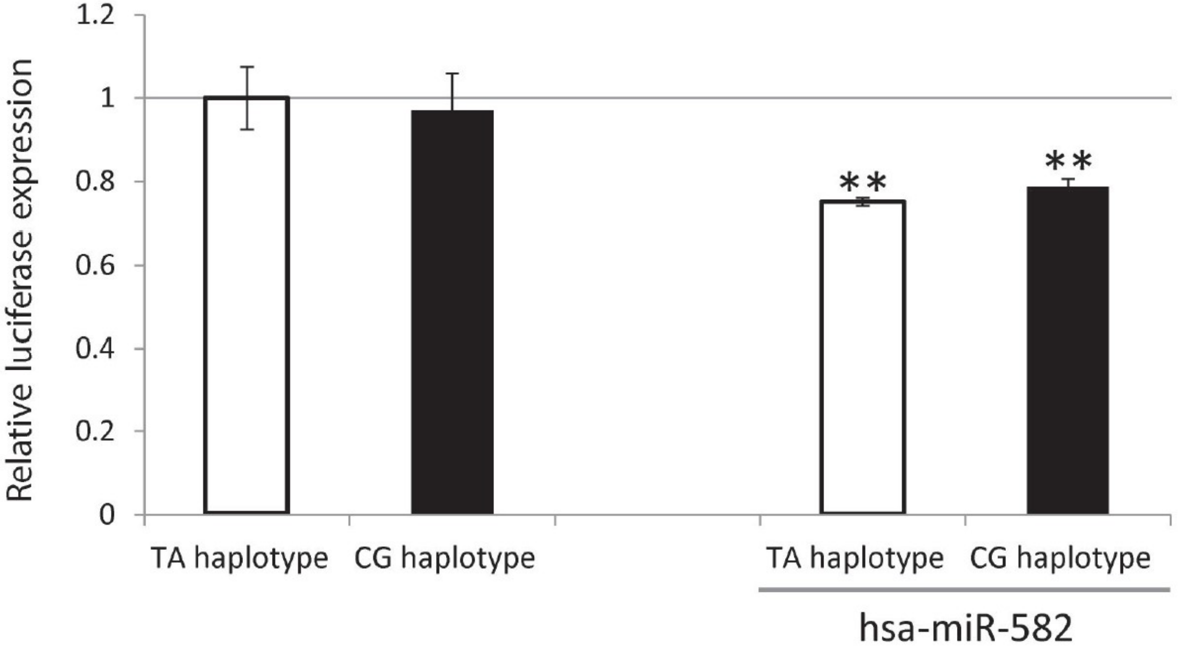

Supplementary Figure 1. Effect of rs708910 on miRNA-based regulation of gene expression. Relative luciferase expression of the pmirGlo vector with the 3'UTR region of *PLCB1* containing the rs1047381-rs708910 T-A and C-G haplotypes in the presence and absence of hsa-miR-582.***p-value*< 0.01

Table S1. SNPs associated with drug dependence in the discovery sample: 735 cases and 739 controls.

		Genotypes (11-12-22)										
		Controls N (%)				Cases N (%)						
**Marker**	**Locus**	**11**	**12**	**22**	**Sum**	**11**	**12**	**22**	**Sum**	**p-value[1]**	**Adj p-value[2]**	**Adj.(OR -95%CI)[2]**
rs6840	*SCD5*	341 (46.5)	317 (43.2)	76 (10.3)	734	284 (39.2)	355 (49.0)	86 (11.8)	725	**0.011**	**0.025**	1.24 (1.03-1.50)
rs1285	*IDI1*	491 (66.6)	231 (31.4)	15 (2.0)	737	535 (72.9)	181 (24.7)	18 (2.4)	734	**0.029**	0.116	1.22 (0.95-1.54)*
rs1872353	*FBXO45*	455 (61.8)	248 (33.7)	33 (4.5)	736	484 (66.4)	222 (30.4)	23 (3.2)	729	**0.044**	0.177	1.16 (0.93-1.45)*
rs6855973	*GRIA2*	483 (65.4)	228 (30.8)	28 (3.8)	739	514 (70.0)	208 (28.4)	12 (1.6)	734	**0.014**	0.114	1.20 (0.95-1.54)*
rs1047383	*PLCB1*	307 (41.6)	339 (45.9)	92 (12.5)	738	269 (36.6)	356 (48.5)	109 (14.9)	734	**0.039**	**9.6e-03**	1.27 (1.06-1.53)
rs1057377	*SPOCK3*	343 (46.5)	329 (44.6)	66 (8.9)	738	393 (53.6)	277 (37.8)	63 (8.6)	733	**0.026**	**0.033**	1.23 (1.02-1.49)*
rs2597775	*QDPR*	341 (46.2)	322 (43.6)	75 (10.2)	738	307 (41.9)	330 (45.1)	95 (13.0)	732	**0.043**	0.439	1.08 (0.89-1.30)

[1]Log-additve model; [2]Ajusted by age; * When OR<1 the inverted score is shown. In bold: nominally significant p-values

Table S2. SNPs associated with drug dependence in the replication sample: 663 cases and 667 controls.

		Genotypes (11-12-22)										
		Controls N (%)				Cases N (%)						
**Marker**	**Locus**	**11**	**12**	**22**	**Sum**	**11**	**12**	**22**	**Sum**	**p-value[1]**	**Adj p-value[2]**	**Adj.(OR -95%CI)[2]**
rs6840	*SCD5*	295 (44.7)	291(44.1)	74 (11.2)	660	295 (44.8)	283 (42.9)	81 (12.3)	659	0.785	0.420	-
rs1285	*IDI1*	492 (74.4)	157 (23.8)	12 (1.8)	661	453 (68.8)	182 (27.7)	23 (3.5)	658	**0.010**	**0.034**	1.32 (1.02-1.71)
rs1872353	*FBXO45*	407 (61.4)	229 (34.5)	27 (4.1)	663	447 (67.6)	186 (28.2)	28 (4.2)	661	0.051	**3.8e-03**	-
rs6855973	*GRIA2*	458 (69.3)	186 (28.1)	17 (2.6)	661	452 (68.5)	191 (28.9)	17 (2.6)	660	0.779	0.486	-
rs1047383	*PLCB1*	284 (42.7)	289 (43.5)	92 (13.8)	665	213 (32.4)	351 (53.3)	94 (14.3)	658	**3.8e-03**	**1.5e-03**	1.37 (1.13-1.67)
rs1057377	*SPOCK3*	349 (52.6)	253 (38.1)	62 (9.3)	664	331 (50.3)	265 (40.3)	62 (9.4)	658	0.516	0.945	-
rs2597775	*QDPR*	293 (44.3)	294 (44.4)	75 (11.3)	662	294 (44.8)	267 (40.6)	96 (14.6)	657	0.461	0.794	-

[1]Log-additve model; [2]Ajusted by age; In bold: nominally significant p-values

Table S3. SNPs associated with drug dependence in the pooled sample: 1393 cases and 1406 controls.

		Genotypes (11-12-22)										
		Controls N (%)				Cases N (%)						
**Marker**	**Locus**	**11**	**12**	**22**	**Sum**	**11**	**12**	**22**	**Sum**	**p-value[1]**	**Adj p-value[2]**	**Adj.(OR -95%CI)[2]**
rs1285	*IDI1*	983 (70.3)	388 (27.8)	27 (1.9)	1398	988 (71)	363 (26.1)	41 (2.9)	1392	0.857	0.754	-
rs1047383	*PLCB1*	591 (42.1)	628 (44.8)	184 (13.1)	1403	482 (34.6)	707 (50.8)	203 (14.6)	1392	**4.8e-04**	**3.7e-04**	1.26 (1.07-1.48)

[1]Log-additve model; [2]Ajusted by age; In bold: nominally significant p-values.
